# Activin receptor-like kinase 1 is associated with immune cell infiltration and regulates *CLEC14A* transcription in cancer

**DOI:** 10.1007/s10456-018-9642-5

**Published:** 2018-08-21

**Authors:** Matteo Bocci, Jonas Sjölund, Ewa Kurzejamska, David Lindgren, Nour-Al-Dain Marzouka, Michael Bartoschek, Mattias Höglund, Kristian Pietras

**Affiliations:** 1 Division of Translational Cancer Research, Department of Laboratory Medicine, Lund University, Medicon Village, Building 404:A3, 223 81 Lund, Sweden; 2 Unit of Urothelial Cancer Genomics, Department of Oncology and Pathology, Lund University, Scheelevägen 8, 22363 Lund, Sweden

**Keywords:** Angiogenesis, Endothelial cell, ALK1, Cell signaling, Pathophysiology, Tumor biology

## Abstract

Cancer cells sustain their metabolic needs through nutrients and oxygen supplied by the bloodstream. The requirement for tumor angiogenesis has been therapeutically exploited in the clinical setting mainly by means of inhibition of the vascular endothelial growth factor family of ligands and receptors. Despite promising results in preclinical models, the benefits for patients proved to be limited. Inadequate efficacy similarly halted the development of agents impinging on the activity of the activin receptor-like kinase (ALK)1, a member of the transforming growth factor-β superfamily. Notwithstanding its characterization as an endothelial cell marker, the full spectrum of biological processes associated with ALK1 is essentially unexplored. Here, we present data revealing the genetic network associated with *ACVRL1* (the gene encoding for ALK1) expression in human cancer tissues. Computational analysis unveiled a hitherto unknown role for *ACVRL1* in relation to genes modulating the functionality of the immune cell compartment. Moreover, we generated a signature of 8 genes co-expressed with *ACVRL1* across different tumor types and characterized the c-type lectin domain containing protein (*CLEC*)*14A* as a potential downstream target of *ACVRL1*. Considering the lack of reagents for ALK1 detection that has hampered the field to date, our work provides the opportunity to validate the 8-gene signature and CLEC14A as biomarkers for ALK1 activity. Ultimately, this may help revisit the clinical development of already existing ALK1-blocking compounds as precision medicines for cancer.

**Electronic supplementary material:**

The online version of this article (10.1007/s10456-018-9642-5) contains supplementary material, which is available to authorized users.

## Introduction

The concept of tumor angiogenesis refers to the ability of a nascent tumor mass to promote vascularization in order to sustain its growth and survival [1]. A fundamental outcome of this proposition is that by inhibiting the release of these factors, tumor development and hematogenous dissemination of tumor cells could be blocked, in practice setting the basis for anti-angiogenic therapy. Indeed, the presence of a vascular network to support the metabolism of cancer cells and to allow their spread to distant organs is a hallmark of solid malignancies [2]. In 2004, the American Food and Drug Administration (FDA) approved the clinical use of bevacizumab, a humanized monoclonal antibody against vascular endothelial growth factor (VEGF)-A, in combination with standard chemotherapy in patients with metastatic colorectal cancer. Unfortunately, the use of this agent showed limited efficacy in breast cancer when administered together with the chemotherapeutic agent paclitaxel: despite almost doubling the progression free survival compared to paclitaxel alone (11.8 vs. 5.9 months), addition of bevacizumab did not extend the overall survival in patients (26.7 vs. 25.2 months) [3]. Experimental studies later uncovered the shortcomings of such type of treatment, i.e., a response phase followed by adaptation to the therapy and bypass of the inhibition [4, 5]. In fact, different modalities to overcome the blockade of angiogenesis are now recognized, from upregulation of pro-angiogenic factors to sprouting angiogenesis, vasculogenesis, intussusception, vessel cooption, vascular mimicry, and cancer stem cell-to-endothelial cell differentiation [6].

In parallel to the increasing realization of inadequate efficacy of VEGF-targeted agents, the search for alternative pathways that regulates neo-angiogenesis ensued. In this context, considerable attention has been given to ALK1. ALK1 is a type I receptor of the TGF-β superfamily and mediates bone morphogenetic protein (BMP)9- and BMP10-induced signaling in the endothelium via the downstream mediators SMAD1/5/8 to orchestrate the development of blood vessels [7]. Despite that ALK1 inhibitors exhibited promising results in a range of different mouse models of cancer [8–11], clinical trials with the receptor decoy dalantercept (Acceleron Pharma) failed to show substantial benefit in different cancer types [12, 13]. One of the major limitations of the drug development has been the absence of validated predictive biomarkers for ALK1 activity in cancer. Also, despite the increasing knowledge about the role of ALK1 in endothelial cell biology, the functional gene network acting downstream of ALK1 remains largely elusive, precluding informed predictions about suitable partners in combinatorial treatment regimens involving ALK1 blockade.

Here, we provide insights to the broader regulatory network associated with *ACVRL1* expression in different human cancers. By interrogating publicly available data on gene expression, we reveal a previously unidentified association between *ACVRL1* and genes controlling immune cell function. Moreover, analysis of the conserved set of *ACVRL1*-correlated genes in 14 different tumor types highlighted an 8-gene signature indicative of ALK1 activity. The gene with the highest median co-expression coefficient across all cancers is *CLEC14A*, which we infer to be a potential direct transcriptional target of ALK1 signaling through SMAD1/5. Taken together, our work prompts further validation of the use of CLEC14A as a surrogate marker for ALK1 activity to guide precision anti-angiogenic therapy in patients, possibly in combination with immunotherapy.

## Materials and methods

### Cell culture, in vitro stimulation, RNA extraction, and qPCR

Mouse endothelial MS1 cells were maintained in culture in DMEM (Invitrogen) supplemented with 10% FCS, penicillin, and streptomycin, in a humidified incubator at 37 °C and 5% CO2. Cells were seeded in 6-well plates at a density of 3 × 105 cells/well and cultured overnight. Next, cells were starved in serum-free medium for 5 h, and further cultured as non-treated, BMP-9-treated, or TGFβ-treated (50 ng/ml and 10 ng/ml, respectively; R&D Systems) in serum-free conditions for 24 h. All experiments were performed in triplicate wells for each condition. Subsequently, cells were washed with PBS, trypsinized, and collected as pellets, which were lysed in RLT buffer. RNA isolation was performed with the RNeasy Mini Kit (Qiagen) according to the manufacturer’s protocol. 0.5 µg total RNA was subsequently reverse-transcribed to cDNA using the iScript cDNA Synthesis Kit (Bio-Rad). 1 µl of the template was used for qPCR (Quant Studio 7 Flex Thermo Fisher Scientific). Expression levels were calculated relative to expression of the reference ribosomal gene RPL19, as calculated by the formula 100*2^− ΔCt^. Primer sequences (forward and reverse, respectively, Invitrogen) for the specific targets were as follows:

- *Rpl19* (GGTGACCTGGATGAGAAGGA, TTCAGCTTGTGGATGTGCTC);
- *Id1* (GAGTCTGAAGTCGGGACCAC, TTTTCCTCTTGCCTCCTGAA);
- *Id3* (ACTCAGCTTAGCCAGGTGGA, GTCAGTGGCAAAAGCTCCTC);
- *Pai1* (TGCATCGCCTGCCATT, CTTGAGATAGGACAGTGCTTTTTCC);
- *Pdgfb* (CCTCGGCCTGTGACTAGAAG, CCTTGTCATGGGTGTGCTTA);
- *Clec14a* (TGGCCAGGTCAGGTCTATGA, CAGGGGGCGAAGATGTGTAG).

### Patient consent, RNAscope, and imaging

Tissue samples were provided by the Sweden Cancerome Analysis Network - Breast: Genomic Profiling of Breast Cancer (SCAN-B) consortium (Permit DNR 2009/658 approved by the national Ethical review board). Patients were enrolled in the clinical trial with the Identifier NCT02306096. Clinical and/or personal data connected to the tissue sample were not disclosed. Informed consent was obtained from all individual participants included in the study. This article does not contain any studies with animals performed by any of the authors.

Tumor pieces from breast cancer patients were directly obtained from surgery and were fresh-frozen in optimum cutting temperature (OCT) cryomount medium (Histolab). 5-µm-thick sections were used for RNAscope detection, following an optimized version of the RNAscope Fluorescent Multiplex Assay protocol (Advanced Cell Diagnostics, ACD). Briefly, sections were fixed in ice-cold 4% fresh paraformaldehyde, for 30 min on ice, washed with PBS, and dehydrated to 100% ethanol. Samples were pre-treated with Protease III, for 30 min at room temperature, followed by probe hybridization (Hs-ACVRL1, #55922; custom-made Hs-CLEC14A-C2, based on #510761) and four canonical steps of amplification at 40 °C, to allow for the appropriate detection of fluorescent signals. Sections were washed and mounted with ProLong Gold anti-fade mounting medium with DAPI (Thermo). The ACD 3-plex negative control probe for channels 1, 2, and 3 was used to determine the specificity and background of the signal. Images were acquired with a LSM 710 laser scanning microscope (Zeiss). At least 4 fields of 3 individual human samples were used for the quantification.

### Gene set enrichment analysis, mutation profiling, and conserved gene signature

The lists of genes co-expressed with *ACVRL1* were obtained by enquiring the “cBioPortal for cancer genomics” [14] in selected provisional studies of The Cancer Genome Atlas (TCGA) repository and the glioblastoma cohort reported in 2013 [15]. Gene ranking was based on Pearson’s R coefficient. RNK files were generated from co-expression data from the cBioPortal and used as ranked list inputs for gene set enrichment analysis (GSEA) preranked analysis (“Hallamarks” gene matrix database, 1000 permutations). In order to obtain a signature of ACVRL1-coexpressed genes conserved across tumor types, the intersection of the different ranked gene lists was calculated with the online tool found at http://bioinformatics.psb.ugent.be/webtools/Venn/.

### TCGA data acquisition and analysis

TCGA RNA-Seq upper quantile normalized FPKM gene expression data and masked Affymetrix SNP 6.0 segmented copy number profiles were downloaded from the Genomic Data Commons (GDC) Data Portal by November 2016. Only primary tumor and normal tissue sample data were used in downstream analyses. In total, 10397 samples from 32 different TCGA projects were included, 21 of which had matching normal tissue samples. For gene expression data, log2 RNA-Seq upper quantile normalized FPKM expression values were calculated after adding an offset of 10^5^. Matched RNA-Seq and copy number profiles were available for 492 primary prostate tumors. Copy number segments less than 10 probes were removed and neighboring segments with log2 fold differences < 0.075 were merged into continuous segments. Plots were produced in R using the “ggplot2” package.

For gene expression data of the BLCA cohort, log2 values of the normalized RNA-seq by expectation maximization (RSEM) counts were downloaded from UCSC Xena hub. The dataset (n = 407 samples) was median re-centered. Samples were classified according to Lund taxonomy classification [16]. The mutation and copy number data for the BLCA cohort were downloaded from Broad Institute of MIT and Harvard. Gene expression-based quantification of immune and stromal cell abundance was carried out using the Microenvironment Cell Populations-counter package [17] in R.

### ChIP-Seq datasets analysis

Feature tracks from previously published ChIP-seq data [18] were visualized with the Integrative genomics viewer (IGV).

To identify transcription factors binding to the CLEC14A DNA region, data from 7353 transcription factor (TF) ChIP-Seq experiments in 31,081 different cell and tissue types were obtained from the ChIP-Atlas database. Thresholds for TF binding were set to ± 5 kb relative to the transcription start site of CLEC14A.

### Statistical analysis

All measurements are depicted as mean ± standard deviation (SD), and statistical analyses were performed using an unpaired two-tailed Student’s *t* test, either with R software or with GraphPad Prism 7. Statistical significance was considered using *α* = 0.05.

## Results

### Components of the ALK1 receptor are a common feature of solid malignancies

**Fig. 1:**
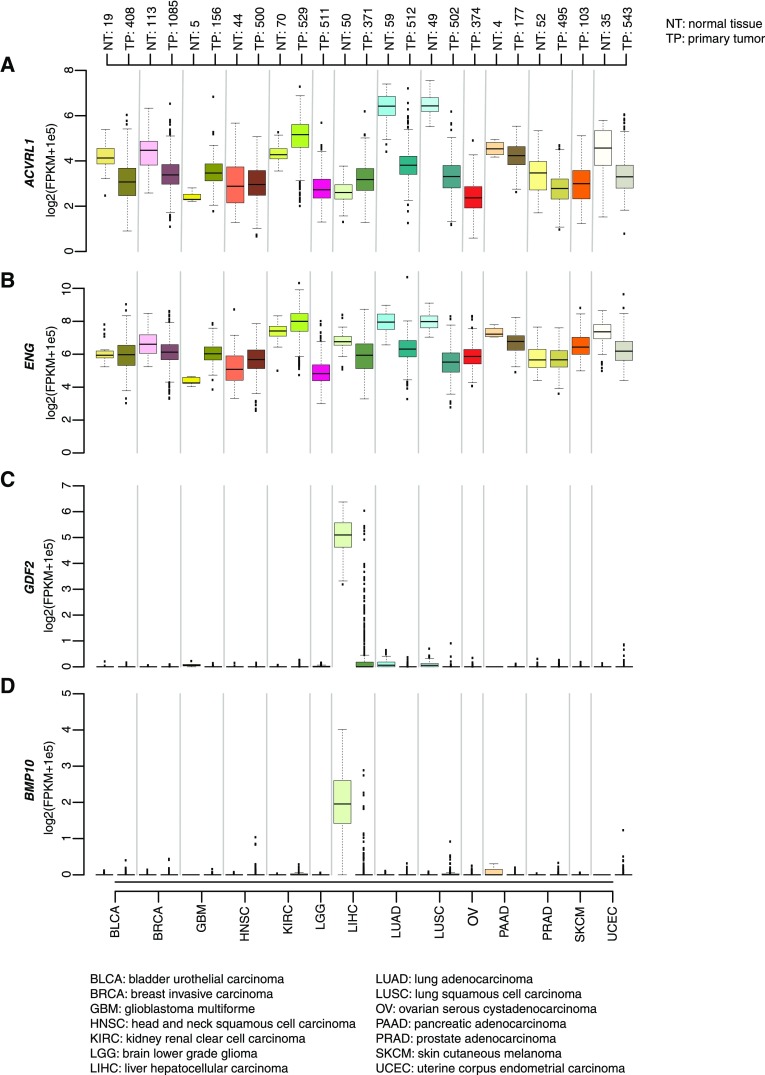
Components of the ALK1 receptor are a common feature of solid malignancies. Box plots of **a***ACVRL1*, **b***ENG*, **c***GDF2*, and **d***BMP10* expression in 14 primary tumor types (TP) and corresponding normal tissue (NT) obtained from the cancer genome atlas (TCGA) repository. The boxes are delimited by the first and third quartile, respectively, whereas the thick lines show the median expression. Outliers exceeding the minimum and maximum of each distribution are depicted as black squares. The number of cases in PT and NT (where available) is indicated above each cohort. FPKM: fragment per kilobase of transcripts per million mapped reads

Given the limited benefit shown by ALK1-blocking agents in clinical trials, we asked whether the expression status of the components of the signaling complex centered around *ACVRL1* delineates tumor types that would benefit from ALK1 inhibition. To this end, expression levels for the receptor *ACVRL1* and the coreceptor endoglin (*ENG*), as well as *GDF2* and *BMP10* (encoding BMP9 and BMP10, the high-affinity ligands for ALK1), were assessed in a panel of 14 cancer types included in the TCGA repository. All normal tissue (NT) counterparts displayed varying levels of expression for *ACVRL1* and *ENG* (Fig. 1a and b). Interestingly, the highest abundance of transcripts for both *ACVRL1* and *ENG* was observed in healthy lungs, consistent with the notion that ALK1 and endoglin are preferentially expressed in capillaries and arterioles of the lungs in adults [19]. Notably, almost all primary tumor (TP) types showed generally reduced levels of both *ACVRL1* and *ENG* compared to NT. In contrast, higher expression of *ACVRL1* was a distinguishing feature of glioblastoma multiforme (GBM) and clear cell renal carcinoma (KIRC). Conspicuous levels of *GDF2* were observed in healthy hepatic tissue, in agreement with a report that identified the liver as the primary source of circulating BMP9 [20] (Fig. 1c). The amount of *BMP10* transcripts was also elevated in the liver, despite the fact that synthesis of this secreted factor is usually limited to the right atrium in adult healthy hearts [21] (Fig. 1d).

### Expression of *ACVRL1* reflects the vascular nature of ALK1

**Fig. 2:**
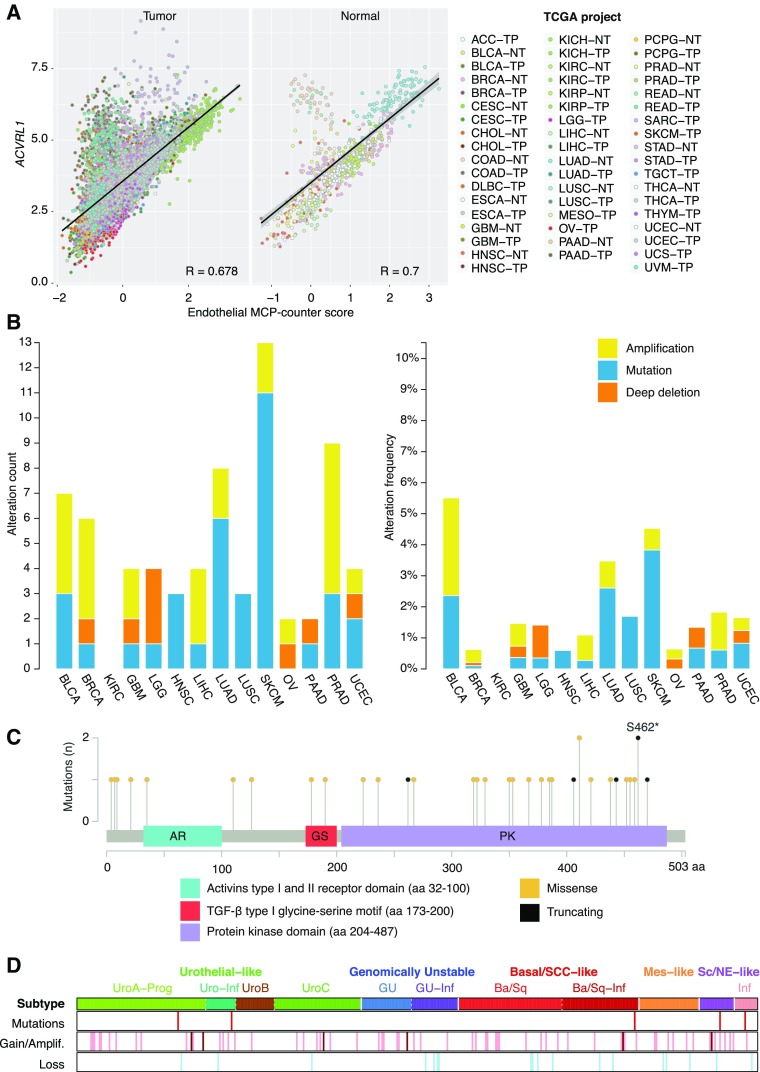
Expression of *ACVRL1* reflects the vascular nature of ALK1. **a** Bar graphs showing absolute count (left) and frequency (right) of different genetic alterations of the *ACVRL1* gene in 14 different tumor types obtained from the cBio portal for cancer genomics. Visualization of **b** point mutations within the different domains of the *ACVRL1* aminoacid sequence from the cBio portal for cancer genomics. **c***ACVRL1* non-silent mutations and copy number alterations in the TCGA bladder cancer (BLCA) cohort. Samples are grouped according to Lund taxonomy classification^15^. *Basal/SCC-like* Basal/Squamous Cell Carcinoma like, *Mes-like* mesenchymal-like, *Sc/NE-like* small-cell/neuroendocrine-like, *Ba/Sq* Basal/Squamous-like, *GU* genomically unstable, *Uro* urothelial-like, *UroA-Prog* urothelial-like A progressed, *Uro-Inf* infiltrated. Red: mutation; pink: gain; dark brown: amplification; light blue: loss. **d** Expression of *ACVRL1* from pan-TCGA data against the endothelial cell microenvironment cell population (MCP)-counter score. For a complete list of common TCGA abbreviations, refer to https://tcga-data.nci.nih.gov/docs/publications/tcga/?

In order to confirm that the expression pattern of *ACVRL1* was mainly restricted to the endothelium, we made use of the microenvironment cell population (MCP)-counter [17], a computational approach developed to estimate the abundance of different subpopulations of stromal cells based on gene expression. When this method was applied to the entire collection of the TCGA repository, *ACVRL1* expression levels showed close association to the reference endothelial MCP-counter score in both normal and tumor tissues, with R equal to 0.7 and 0.678, respectively (Fig. 2a). Of note, healthy tissue originating from colon and rectum showed a higher relative expression of *ACVRL1* transcripts over estimated endothelial cell content. In tumor samples, tissues from different origins displayed generally more heterogeneous levels of *ACVRL1* in endothelial cells (Fig. 2a). The expression of *ACVRL1* did not show any correlation with immune cell subsets or with fibroblasts in healthy (R between 0.261 and 0.374) or tumor (R between 0.197 and 0.374) specimens.

Tumor cells acquire properties that confer growth advantage over the surrounding healthy cells through genetic modifications such as mutations. To investigate whether any tumor types might be fueled by ectopic ALK1 expression in malignant cells, we analyzed the incidence of different types of mutations and their distribution within the coding sequence of the *ACVRL1* gene. Except for KIRC, the remaining tumor types all showed a broad range of *ACVRL1* mutations. Lung adenocarcinoma (LUAD), malignant melanoma, and prostate carcinoma were the three cancer types with highest mutation count (Fig. 2b, Supplementary Tables 1 and 2). Nevertheless, bladder cancer (BLCA) was the tumor type with the highest alteration frequency (Fig. 2b). Out of the total 38 point mutations identified, the majority (73,7%) was found within the *ACVRL1* kinase domain (PK), followed by the glycine-serine (GS)-rich domain (5,3%), whereas only one mutation (2,6%) was detected in the activin receptor (AR) domain (Fig. 2c). Seven additional missense mutations (18,4%) affected residues outside the above-mentioned domains of ALK1 (Fig. 2c). Moreover, BLCA displayed the highest frequency of *ACVRL1* amplification (Supplementary Table 1). We then asked whether these *ACVRL1*-amplified tumors would show specific characteristics compared to the rest of their respective groups. Evaluation of the copy number variation in BLCA indicated that these patients were equally represented in the different subtypes of this disease [16, 22] (Fig. 2d). In conclusion, these results suggest that the observed *ACVRL1* genetic lesions in epithelial cells most likely signify passenger events and not driver mutations.

### Both novel and established biological processes are associated with *ACVRL1* in human cancer

**Fig. 3:**
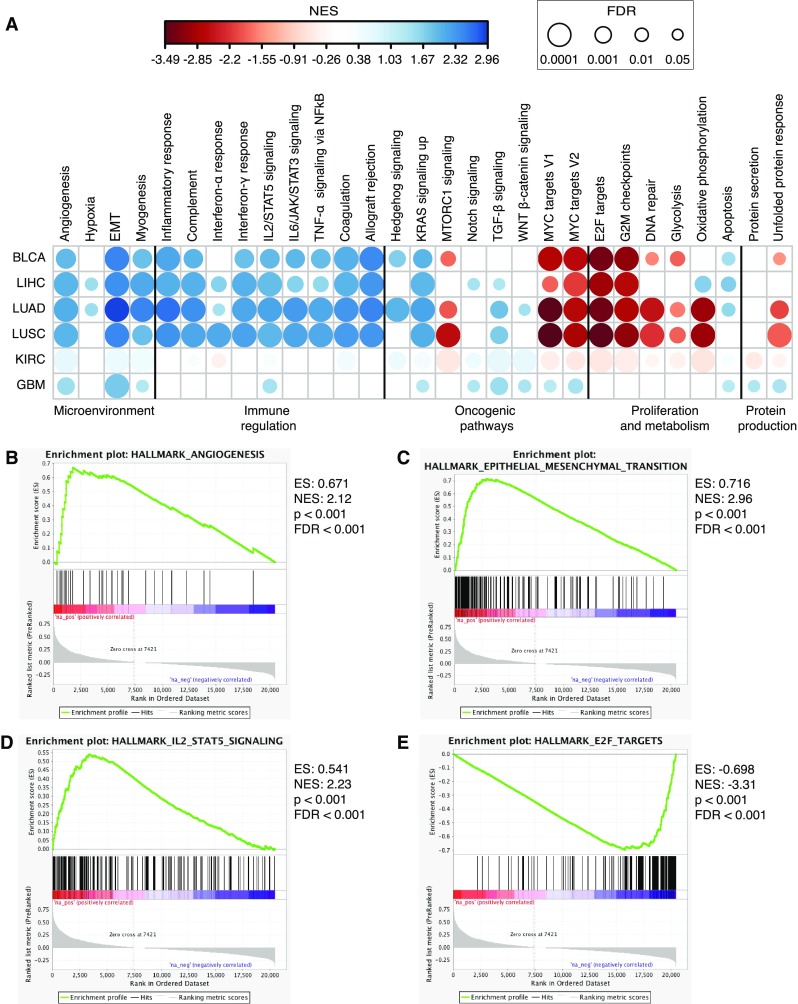
Processes affecting the properties of the tumor mass are associated with *ACVRL1* in human lung cancer. **a** Bubble matrix depicting gene set enrichment analysis (GSEA) plots of the genes associated with *ACVRL1* in the TCGA BLCA, LIHC, LUAD, LUSC, KIRC, and GBM cohorts. The matrix simultaneously illustrates NES (color) and FDR values adjusted for multiple testing (size). Gene lists were ranked based on Pearson’s R coefficient in the cBio portal for cancer genomics. Representative enrichment plots for the GSEA in the LUAD cohort: **b** angiogenesis, **c** epithelial-to-mesenchymal transition, **d** IL2 STAT5 signaling, and **e** E2F targets. *ES* enrichment score, *NES* normalized enrichment score, *p* nominal *p* value, *FDR* false discovery rate *q* value

In order to gain a broader understanding of the gene network linked to *ACVRL1* in human cancers, a ranked list of *ACVRL1*-correlated genes was generated in the cBioPortal for cancer genomics and subjected to gene set enrichment analysis (GSEA), using the “hallmarks” collection as reference sets [23, 24]. We selected the bladder (BLCA), liver (LIHC), and lung (adenocarcinoma LUAD and squamous cell carcinoma LUSC) cohorts, as well as the renal cell carcinoma (KIRC) and glioblastoma (GBM) datasets for further analysis. All the tumor types shared a core of processes associated to the tumor microenvironment and its composition (Fig. 3a), including angiogenesis (Fig. 3b), EMT (Fig. 3c) and immune regulation (Fig. 3d); strikingly, all the gene sets related to immune function, i.e., “inflammatory response,” “complement,” “interferon-γ,” “IL2/STAT5” and “IL6/JAK/STAT3” signaling, “TNF-α,” “coagulation,” and “allograft rejection,” showed a coherent association with *ACVRL1* expression (Fig. 3a and Supplementary Table 3). Conversely, molecular signatures of cell-cycle modulation, e.g., “E2F targets” and “G2M checkpoints” were negatively correlated to *ACVRL1* (Fig. 3a, e and Supplementary Table 3). Nonetheless, the tumor types with highest expression of *ACVRL1*, namely KIRC and GBM, displayed GSEA profiles that were distinct from those of the remaining cohorts included in this investigation; KIRC showed negative enrichment scores for all metabolic processes and protein production, whereas GBM only showed positive enrichment for all the reference hallmarks (Fig. 3a and Supplementary Table 3).

In conclusion, expression of *ACVRL1* is not simply coupled to established pathways such as angiogenesis, but is also extended to a regulatory network of processes that affect both malignant cells and other cellular entities of the tumor microenvironment, specifically immune cells.

### A set of 8 genes co-expressed with *ACVRL1* is conserved in different tumor types

Next, ranked lists of *ACVRL1*-correlated genes with a Pearson coefficient ≥ 0,5 were generated and used to determine the commonality of gene regulation instigated by *ACVRL1* expression across tumor types. Comparative analysis of the lists led to an 8-gene signature, the expression of which was conserved in all malignancies included in the present investigation (Supplementary Tables 4 and 5). Five of the genes comprised in the list were intimately related to endothelial cell function, including roundabout guidance receptor (*ROBO*)*4*, endoglin (*ENG*), platelet and endothelial cell adhesion molecule (*PECAM*)*1*, and protocadherin (*PCDH*)*12*. Interestingly, the gene with the highest median co-expression coefficient in the set was *CLEC14A*, recently characterized as a tumor-specific endothelial marker [25]. Co-expression of *ACVRL1* and the low-affinity receptor for interleukin 3 (*IL3RA*) and the G protein-coupled receptor (*GPR*)*4* was also preserved in the different tumor types. The last member of this gene list was the Chromosome X open reading frame 36 (CXorf36), for which there is only limited annotation available on biological relevance and function.

In order to infer knowledge about the function of the co-expressed genes, the 8 conserved candidates were used as input for the web-based tools for enrichment analysis, i.e., TOPPgene [26] and Enrichr [27]. As expected, our gene set was significantly enriched in ontology terms related to biological processes like “angiogenesis,” “blood vessel development,” and “vascular development,” as well as for the mammalian phenotype “decreased angiogenesis” and “abnormal blood vessel” (Table 1). Reassuringly, and in agreement with the generation of our list from cBioPortal cancer genomics data, “tumor angiogenesis” and “tumor vasculature” were the most significant sets in the category “disease” (Table 1).

**Table 1:** Gene ontology terms and processes enriched with the *ACVRL1* signature

Category	Description	*p* value	Bonferroni *q* value	Hit in query list
Biological process	GO 0001525: angiogenesis	2.459E-5	9.64E-03	ENG,PECAM1,ROBO4,GPR4
	GO 0048514: blood vessel morphogenesis	4.827E-5	1.89E-02	ENG,PECAM1,ROBO4,GPR4
	GO 0001568: blood vessel development	9.257E-5	3.63E-02	ENG,PECAM1,ROBO4,GPR4
	GO 0001944: vascular development	1.078E-4	4.23E-02	ENG,PECAM1,ROBO4,GPR4
Co-expression	20421987-Table S1	1.532E-6	1.27E-03	ENG,PECAM1,ROBO4,CLEC14A,GPR4
Co-expression Atlas	JC_hmvEC_1000_K4	1.123E-11	6.05E-09	ENG,ROBO4,IL3RA,CLEC14A,GPR4 PCDH12,CXorf36
	JC_hmvEC_500_K1	3.379E-11	1.82E-08	ENG,ROBO4,CLEC14A,GPR4 PCDH12,CXorf36
	JC_hmvEC_2500_K1	2.672E-9	1.44E-06	ENG,ROBO4,IL3RA,CLEC14A,GPR4 PCDH12,CXorf36
	PCBC_ctl_PulmonMicrovasc_1000	3.731E-9	2.01E-06	ENG,ROBO4,IL3RA,CLEC14A,GPR4 PCDH12,CXorf36
	PCBC_ctl_CardioEndothel_1000	3.757E-9	2.03E-06	ENG,ROBO4,IL3RA,CLEC14A,GPR4 PCDH12,CXorf36
	JC_hmvEC_1000	4.003E-9	2.16E-06	ENG,ROBO4,IL3RA,CLEC14A,GPR4 PCDH12,CXorf36
	PCBC_ratio_CardioEndothel_vs_SC_cfr-2X-p05	2.389E-7	1.29E-04	ENG,ROBO4,IL3RA,CLEC14A,GPR4 PCDH12,CXorf36
	gudmap_RNAseq_e15.5_Endothelial_2500	2.763E-7	1.49E-04	ENG,PECAM1,ROBO4,CLEC14A,GPR4 PCDH12,CXorf36
Disease	C1658953: tumor vasculature	2.558E-5	9.13E-03	ENG,PECAM1,CLEC14A
	C1519670: tumor angiogenesis	6.184E-5	2.21E-02	ENG,PECAM1,ROBO4,GPR4
Mammalian phenotype	MP0001614: abnormal blood vessel	1.40E-04	9.26E-03	ENG, PECAM1, ROBO4, GPR4
	MP0005602: decreased angiogenesis	1.73E-06	1.54E-04	ROBO4;GPR4;ENG

**Fig. 4:**
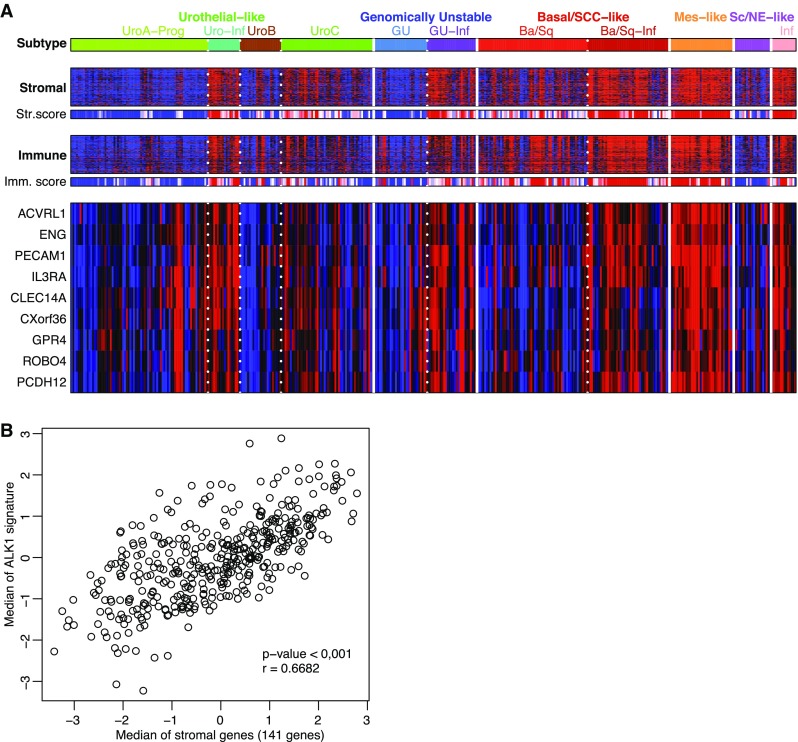
A set of 8 genes conserved across tumor types and associated with *ACVRL1* are indicative of stromal and immune cell infiltration in bladder cancer. **a** Expression of *ACVRL1* and the 8-gene signature in the different subtypes of the TCGA BLCA cohort (grouping and subtype abbreviations as in Fig. 2). Vertical lines separate major molecular subtypes, whereas dotted lines separate subgroups of subtypes. Stromal and immune gene expression signatures based on previously published data^28^. Stromal and immune scores show the tumor purity scores based on the ESTIMATE tool^16^. *ACVRL1* activin receptor-like kinase 1, *ENG* endoglin, *PECAM1* platelet and endothelial cell adhesion molecule 1, *IL3RA* interleukin 3 receptor subunit alpha, *CLEC14A* C-type lectin domain containing 14A, *CXorf36* chromosome X open reading frame 36, *GPR4* G protein-coupled receptor 4, *ROBO4* Roundabout guidance receptor 4, *PCDH12* protocadherin 12. **b** Pearson’s correlation between stromal signature^28^ and the *ACVRL1* gene signature generated in the current investigation

Since the microenvironment is able to influence the growth and progression of a neoplastic lesion, we wondered if our condensed gene list could be informative of tumor composition when applied to a recently improved classification of urothelial cancer [22]. Interestingly, the signature showed coordinate expression across the bladder cancer data set: indeed, the signature exhibited high expression in tumors infiltrated by stroma and immune cells (Fig. 4a). Additionally, the highest expression was noted in the mesenchymal-like subtype, a group of tumors that has likely undergone EMT given the expression of genes such as *VIM* and *ZEB2* [22] (Fig. 4a). Finally, the 8-gene signature also showed good concordance with the stromal signature developed to estimate the level of infiltration in bladder tumors [28] (Fig. 4b).

In conclusion, more than simply consolidating the role of ALK1 as a regulator of endothelial cell activity, the set of *ACVRL1*-co-expressed genes can be employed as a proxy for stromal infiltration in cancer.

### Expression of CLEC14A is regulated by TGF-β superfamily signaling

Within the 8-gene signature, *CLEC14A* was the gene with the highest ranking of co-expression with *ACVRL1*. Hence, *CLEC14A* was selected for downstream analysis. Prior to its suggested role in tumor angiogenesis, CLEC14A was described as an endothelial-specific adhesion molecule [29] and further molecular characterization detected high levels of CLEC14A in the brain, retina, lungs, lymph nodes, ears, blood and lymphatic vessels of mice [30].

First, we examined the dataset in which *CLEC14A* was initially identified as a differentially expressed gene during the transition from progenitor to endothelial lineage-committed cells [31]. Inspection of this gene list revealed that in accordance with increased *CLEC14A* expression, *ACVRL1, ENG*, and the coreceptor *TGFBR2* were all upregulated through this process, whereas *TGFBR1* was downregulated (data not shown).

**Fig. 5:**
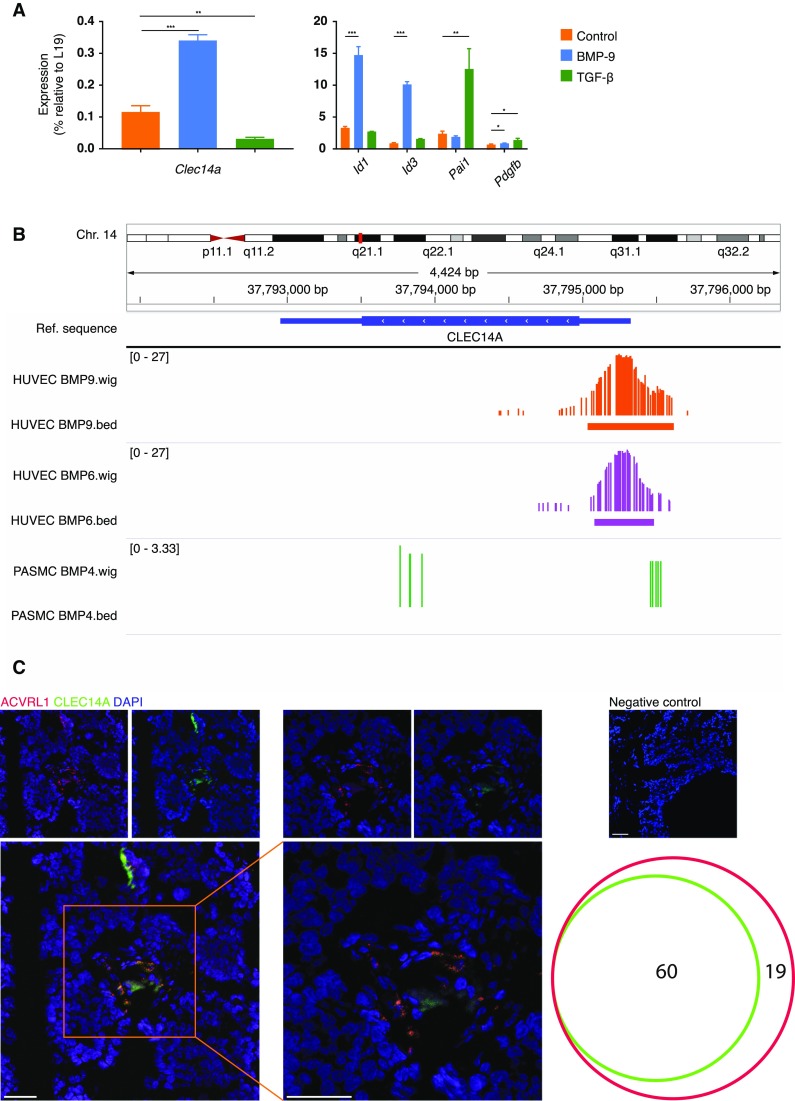
*ACVRL1* directly regulates the transcription of *CLEC14A*. **a** Quantitative reverse transcription polymerase chain reaction (qRT-PCR) expression levels of *Clec14a* transcripts in murine endothelial MS1 cells following stimulation with recombinant TGF-β and BMP-9 (both at 5 ng/ml) for 72 h. ***p < 0.001. The graph shows the average of three biological replicates. **b** Integrative genomics viewer browser visualization of the feature tracks of chromatin immunoprecipitation (ChIP)-seq data^17^ of human umbilical vein endothelial cells (HUVECs) stimulated with either BMP6 or BMP9 and placental arterial smooth muscle (PASM) cells stimulated with BMP4. The peaks correspond to SMAD1/5 binding to *CLEC14A*. **c** Dual RNAscope in situ hybridization of human breast cancer samples. Upper panels: individual channels for *ACVRL1* (red) and *CLEC14A* (green). Blowup: co-expression of *ACVRL1* and *CLEC14A*. Cell nuclei were counterstained with 4′,6′-diamidino-2-phenylindole, dihydrochloride (DAPI) (blue). Scale bars: 50 µm. The Venn diagram shows the quantification of each probe on DAPI-positive foci in a total of 14 optical fields from three individual human samples

Next, we set out to confirm that the TGF-β superfamily could regulate the expression of *CLEC14A*. To this end, murine endothelial MS1 cells were stimulated *in vitro* with either recombinant TGF-β1 or BMP9, for 24 h. Analysis of *Clec14a* transcripts by qPCR highlighted a dual regulation, with a significant upregulation following BMP9 stimulation and a significant downregulation upon TGF-β supplementation in the culture medium (Fig. 5a).

### SMAD1/5 directly bind *CLEC14A* promoter

As transcriptional regulation can be both a direct and a mediated event, we sought to determine how *ACVRL1* controlled the expression of *CLEC14A*. To assess a direct ability of *ACVRL1* transcriptional downstream regulators SMAD1/5 to access the promoter region of *CLEC14A*, we interrogated available ChIP-seq data from BMP-stimulated endothelial cell lines [18]. Analysis of human umbilical vascular endothelial cells (HUVECs) stimulated with either BMP9 or BMP6 showed a peak signal corresponding to SMAD1/5 binding to the promoter region of *CLEC14A* (Fig. 5b). Importantly, this peak was not observed in Placental Arterial Smooth Muscle (PASM) cells stimulated with BMP4, indicating that despite a common downstream activation of SMAD 1/5, both the cell type and the ligand play a pivotal role in the regulation of expression of specific targets (Fig. 5b).

Given the general low DNA-binding affinity of the SMAD factors, the transcriptional machinery downstream of TGF-β superfamily receptors is orchestrated by a multitude of factors that tightly regulate targets in a temporal- and tissue-specific manner. We therefore evaluated the binding of other components to the promoter of *CLEC14A* that could drive its transcription in concert with SMAD1/5. To this end, we employed an unbiased approach by screening available ChIP-seq data on human cells and extracted the factors bound within a ± 5 kb-region around the transcription start site for *CLEC14A*. When restricting our interest to the endothelial cell compartment, together with BRD4 and RELA (encoding for the p65 subunit of NF-κB), the transcriptional coactivator EP300 emerged as a significantly enriched factor bound to *CLEC14A* (Table 2, see experimental IDs in bold).

**Table 2:** Statistically significant transcription factors bound to human *CLEC14A*

ID	Cell class	Cell type	Factor	*p* value	Fold enrichment
SRX317579	Pluripotent stem cell	iPS cells	EZH2	< 0.01	153.3
SRX317594	Pluripotent stem cell	iPS cells	EZH2	< 0.01	108.5
SRX151222	Blood	RS4-11	KMT2A	< 0.05	79.6
SRX317589	Pluripotent stem cell	iPS cells	JARID2	< 0.05	79.3
SRX1127543	Blood	RS4-11	NR3C1	< 0.05	70.8
**SRX425253**	**Cardiovascular**	**HUVEC**	**BRD4**	< **0.05**	**65.3**
SRX317601	Pluripotent stem cell	iPS cells	JARID2	< 0.05	55.9
**SRX1116232**	**Cardiovascular**	**HUVEC**	**EP300**	< **0.05**	**41.9**
SRX317605	Pluripotent stem cell	iPS cells	JARID2	< 0.05	34.6
SRX317609	Pluripotent stem cell	iPS cells	JARID2	< 0.05	34.2
SRX1901489	Blood	CD19 + leukemic cells	MLL-AF4	< 0.05	33.6
**SRX294971**	**Cardiovascular**	**HUVEC**	**RELA**	< **0.05**	**33.2**
SRX317602	Pluripotent stem cell	iPS cells	EZH2	< 0.05	31.1
SRX317606	Pluripotent stem cell	iPS cells	EZH2	< 0.05	28.8
**SRX112016**	**Cardiovascular**	**HUVEC**	**RELA**	< **0.05**	**28.7**
SRX151223	Blood	RS4-11	AFF1	< 0.05	27.0
SRX656346	Blood	ICN12	BCL6	< 0.05	26.3
SRX235030	Blood	NALM-6	IKZF1	< 0.05	22.2
SRX553658	Pluripotent stem cell	hESC H1	TRIM28	< 0.05	21.9
SRX959099	Blood	NALM-6	NR3C1	< 0.05	21.0
SRX317598	Pluripotent stem cell	iPS cells	EZH2	< 0.05	20.7

Experimental conditions restricted to the endothelial cell compartment are shown in bold

To conclusively demonstrate a direct association between *ACVRL1* and *CLEC14A* expression, we performed simultaneous RNAscope in situ hybridization on human breast tumor specimens. Confocal imaging revealed *ACVRL1* single-positive cells, as well as *ACVRL1* and *CLEC14A* double-positive cells (Fig. 5c). Of fundamental importance, we could not observe any *CLEC14A* single-positive cell, reinforcing the hypothesis that *CLEC14A* is strictly associated with ALK1 expression.

## Discussion

Collectively, our study has revealed a broader regulatory network associated with ALK1 activation in cancer. The use of computational analysis enabled the generation of cancer-specific sets of genes associated with *ACVRL1* expression that could be further refined to obtain a single list of common factors conserved across different tumor types. Ultimately, the validation of *CLEC14A* as a transcriptional target of *ACVRL1* for biomarker use, and the regulation of immune response as a process correlated with *ACVRL1* expression may hold utility for re-evaluating the clinical development of already existing ALK1-blocking agents.

Our cross-cancer analysis highlights a variable, but consistent, expression of *ACVRL1* in all tumor types. In particular, the reduced levels of *ACVRL1* compared to the corresponding normal tissues suggest the inability of the proliferating malignant mass to develop a vascular tree to adequately sustain the metabolic needs of the tumor cells. Our data are compatible with findings indicating a specific role for ALK1 in mediating the maturation phase of angiogenesis [32] and are in agreement with the known aberrant nature of tumor-associated vessels. The higher expression of *ACVRL1* in KIRC and GBM tumors might reflect the architecture of the organs in which these cancers arise and develop, including a naturally strict dependency on the vasculature of these tumor types.

In light of the GSEA analysis, the association of *ACVRL1* to processes related to immune cell regulation might constitute a rationale for a combined treatment regimen based on ALK1 inhibition and immunotherapy agents. In support of this hypothesis, ALK1-co-expressed genes were highly enriched in IL2/STAT5 and IL6/JAK/STAT3 pathways; the former has been implicated in the proliferation and development of peripheral T cells and regulatory T cells [33, 34], whereas the latter is a strong and recognized tumor immunosuppressive signaling cascade [35, 36]. In this context, characterization of the immune infiltration will be of paramount importance to determine whether specific tumor types might benefit from combined therapy. Recent work proposes a triggering of the intra-tumoral immune response following vessel normalization induced by anti-VEGF therapy [37, 38]. Intriguingly, an analogous vascular phenotype was reported in different studies that characterized the *in vivo* activity of ALK1-Fc [8, 9, 39, 40].

As already mentioned, the clinical benefit of targeted therapy has been limited by tumor evolution and adaptation to anti-cancer agents. The analysis of the mutational landscape of *ACVRL1* indicated that this locus is affected by somatic alterations with a relatively low frequency. In line with these observations, amplification of *ACVRL1* did not confer tumors the biological advantage typical of a putative oncogenic driver. Interestingly, some of the loss of function mutations observed in tumors are reported to affect the receptor kinase domain (e.g., the missense R411Q [41], and the truncating W406*, S462*, and E470* mutations [42–44]) and have already been described in human hereditary telangiectasia (HHT)2, an autosomal dominant genetic vascular disorder caused by mutations in *ACVRL1*. Again, these events were randomly distributed in the different datasets and did not confer any overt advantage to the tumors.

Our effort to identify a set of common *ACVRL1*-related genes whose expression is preserved across different cancer types confirms the primary role of ALK1 as a mediator of endothelial cell fate. Among the 8 conserved genes across different tumor types, *CLEC14A* was the one with the highest mean co-expression coefficient. CLEC14A was initially described as a fundamental component of the cell-to-cell adhesion machinery [29] and just a year later, a function as a tumor endothelial-specific marker was proposed [25]. The exact role of CLEC14A in angiogenesis is still debated, as two independent studies (based on rather different investigational endpoints) reported opposite effects when knocking out *Clec14a* in a mouse model of lung carcinogenesis [30, 45]. Nonetheless, based on much more similar experimental setups, the reduced sprouting of VEGF-stimulated HUVEC *in vitro*, as well as the reduced tumor volume and associated vascular density *in vivo* reported in *Clec14a* knock-out mice [45], phenocopies the effects of ALK1 inhibition observed in different studies [8, 10, 11]. Similarly, analysis of *Clec14a* expression in a transgenic mouse model of pancreatic neuroendocrine tumorigenesis demonstrated an increased expression only in full-blown tumors with a more mature vessel phenotype, but not in islets that have undergone an angiogenic switch to fuel their proliferation [46], supporting the reports of ALK1 expression in the resolution phase of angiogenesis [7].

As ascertained by ChIP-seq data of human endothelial cells, stimulation with the high-affinity ligand BMP9 produced a strong binding peak of SMAD1 in the promoter region of *CLEC14A*. To confirm this type of regulation, dual RNAscope-ISH on human breast cancer samples unveiled that expression of *ACVRL1* is required for the concurrent detection of *CLEC14A* in the same cell. In conclusion, we propose that *CLEC14A* is under the transcriptional control of *ACVRL1*. The lack of reliable reagents for the detection of ALK1, paired with the paucity of predictive biomarkers for ALK1 blockade, has hampered the translation of ALK1 inhibitors to clinical care. This highlights the need for activity-based assessment of the ALK1 pathway as a predictive biomarker for patient selection. In this context, our 8-gene profile, as well as CLEC14A, might represent a starting point for the development of a companion tool for precision targeting of ALK1-driven tumor angiogenesis.

Furthermore, our results suggest other modalities of *CLEC14A* regulation exerted by *ACVRL1*, bringing together some of the aspects we have already discussed, e.g., the relationship between endothelial ALK1 expression and the modulation of the properties and the composition of the tumor microenvironment. The unbiased assessment of transcriptional regulators bound to the promoter of *CLEC14A* revealed a significant occupancy of the coactivator EP300, an acetyltransferase that orchestrates transcription via chromatin remodeling [47]. Although EP300 is a common cofactor with very broad functions in cell growth and division, the presence of this enzyme is relevant given its ability to cooperate with another enriched factor bound to the promoter region of *CLEC14A*, namely RELA/p65 (encoding for the p65 subunit of NF-κB). Indeed, RELA and EP300 can promote the activation of E-selectin and vascular cell adhesion molecule (VCAM)-1, fundamental mediators of leukocyte adhesion to endothelial cells [48], allowing the extravasation and tissue infiltration steps of the inflammation cascade to further coordinate the immune response. In line with these observations, our results show that genes associated to *ACVRL1* expression are significantly enriched in “TNF-α via NF-κB signaling.” Lastly, the bromodomain containing protein (BRD)4 was the most significantly enriched element bound to *CLEC14A*. Of note, BRD4 and RELA/p65 jointly drive the inflammatory transcriptional response [49], whereas more recently a study focused on the direct interaction between these two proteins following TNF-α stimulation of endothelial cells [50].

In conclusion, our results shed light on previously unknown functional associations elicited by the downstream effectors of ALK1 in endothelial cells. The future validation of our 8-gene signature and CLEC14A as biomarkers to follow the activation status of ALK1, paired with the potential combination of ALK1 inhibitors with immunomodulatory compounds, may motivate reconsideration of the halted clinical development of already existing ALK1-blocking agents.

## Electronic supplementary material

Below is the link to the electronic supplementary material.

Supplementary material 1 (XLSX 13 KB)

Supplementary material 2 (XLSX 12 KB)

Supplementary material 3 (XLSX 21 KB)

Supplementary material 4 (XLSX 12 KB)

Supplementary material 5 (XLSX 3980 KB)
